# Electrophysiology of the rhythmic defecation program in nematode *Heterorhabditis megidis*

**DOI:** 10.1038/s41598-017-18118-y

**Published:** 2017-12-19

**Authors:** Victor P. Kuznetsov, Georgy A. Slivko-Koltchik, Dmitry A. Voronov, Yuri V. Panchin

**Affiliations:** 1 Kharkevich Institute for Information Transmission Problems, Russian Academy of Sciences, Moscow, 127994 Russian Federation; 2 Faculty of Bioengineering and Bioinformatics, Lomonosov Moscow State University, Moscow, 119991 Russian Federation; 3 A.N. Belozersky Institute of Physico-Chemical Biology Moscow State University, Moscow, 119991 Russian Federation

## Abstract

The nervous system controls most rhythmic behaviors, with a remarkable exception. In *Caenorhabditis elegans* periodic defecation rhythm does not appear to involve the nervous system. Such oscillations are studied in detail with genetic and molecular biology tools. The small size of *C*. *elegans* cells impairs the use of standard electrophysiological methods. We studied a similar rhythmic pacemaker in the noticeably larger gut cells of *Heterorhabditis megidis* nematode. *H*. *megidis* defecation cycle is driven by a central pattern generator (CPG) associated with unusual all-or-none hyper-polarization “action potential”. The CPG cycle period depends on the membrane potential and CPG cycling also persisted in experiments where the membrane potential of gut cells was continuously clamped at steady voltage levels. The usual excitable tissue description does not include the endoderm or imply the generation of hyper-polarization spikes. The nematode gut cells activity calls for a reevaluation of the excitable cells definition.

## Introduction

Central pattern generators (CPGs) are cellular networks or single cells that produce rhythmic patterned outputs in isolation from sensory feedback. Cellular and molecular mechanisms of circadian (about 24 hours) and fast (with period of seconds) rhythms are well studied, while less attention has been paid to ultradian rhythms with shorter periods (minutes to hours)^1^. One of these CPGs is a defecation motor program (DMP) in *C*. *elegans*.

**Figure 1:**
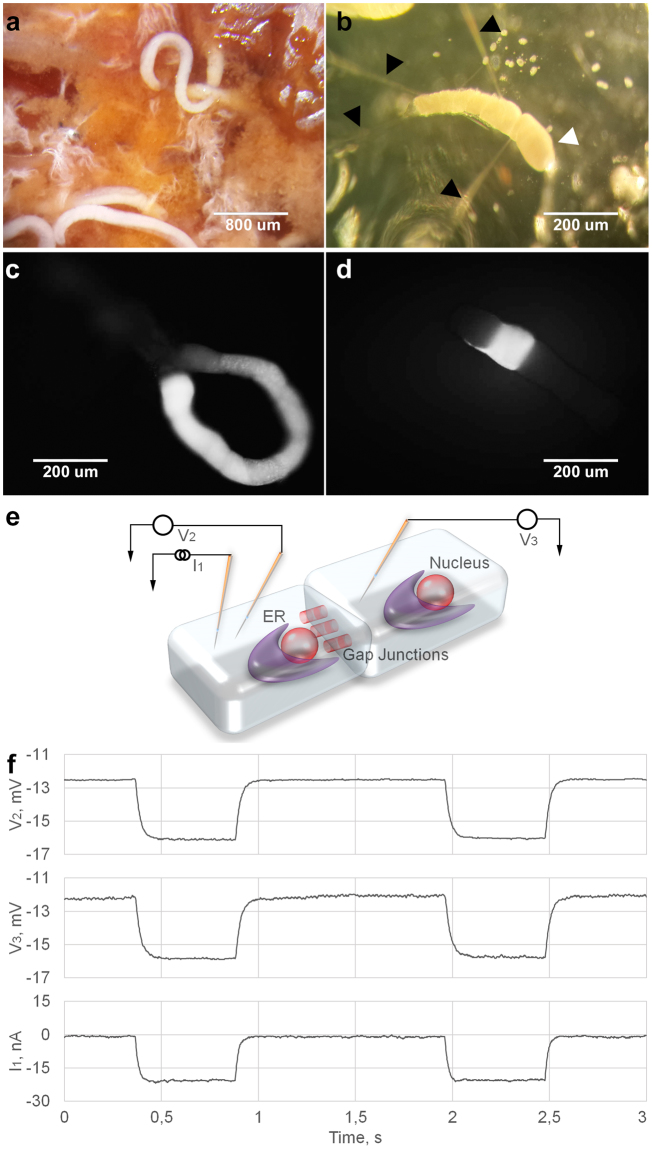
Gap junctions in *H*. *megidis* nematode gut. (**a**) *H*. *megidis* parasites inside a dissected insect. (**b**) Isolated preparation of a nematode gut (white arrowhead) with four intracellular microelectrodes (black arrowheads). (**c**,**d**) Fluorescent dye injections into a single gut cell. **c** Lucifer yellow CH diffuses to adjacent cells within several minutes. (**d**) Carboxyfluorescein retains in one cell after one hour after injection. (**e**) Scheme of experiment, in which two microelectrodes I_1_ and V_2_ are placed in one gut cell; V_3_ electrode is inside the adjacent cell. (**f**) Impulse of negative current of −20 nA is applied through I_1_. Deviation of membrane potentials in adjacent cells ΔV_2_ and ΔV_3_ is registered by V_2_ and V_3_. Coupling coefficient is the ratio ΔV_3_/ΔV_2_.

*C*. *elegans* is one of the best studied model organisms. Molecular and genetic studies of this species started with the works of Sydney Brenner in 1974^2^. An adult *C*. *elegans* hermaphrodite eat nearly continuously in the presence of food, while adult males could leave food in search of hermaphrodites^3^. While eating *C*. *elegans* rhythmically defecating with the stable period of 45–50 seconds. During rest periods/lethargus defecation cycle is much prolonged^4,5^. In addition when animals are not exposed to food the defecation motor program is not executed. The genetics of this process have been studied for quite a long time^6–8^. The posterior body wall muscle contraction (pBoc) in the defecation cycle is regulated by pH changes in the pseudocoelomic space caused by Na^+^/H^+^ transporter activity in intestinal cells^9,10^. It was found that DMP is controlled by intestinal calcium waves^11^. Some studies show an important role of gap junctions (GJ) for calcium wave propagation^12^. In *C*. *elegans* periodic defecation rhythm does not appear to involve the nervous system^13^. Electrophysiological experiments in *C*. *elegans* are difficult because of the small cell sizes in this nematode. *H*. *megidis* (Fig. 1a) is an obligate parasite of insects and belongs to the same nematode order *Rhabditida* as *C*. *elegans*. *H*. *megidis* has noticeably bigger gut cells (Fig. 1b) which may facilitate electro-physiological studies in nematodes, supplement and refine remarkable studies of *C*. *elegans* gut physiology and the DMP.

Using this model we demonstrated that the defecation cycle is driven by a CPG associated with unusual all-or-none hyper-polarization “action potential” with a fixed duration of about one minute, period of about four minutes and amplitude of about 60 mV. The CPG cycle period depends on the membrane potential. A short hyperpolarizing current pulse could shift the cycle phase. This leads to suggestion of plasma membrane voltage gated mechanisms involvement. However, CPG cycling persisted in experiments where the membrane potential of gut cells was continuously clamped at steady voltage levels indicating that intracellular mechanisms are also involved. Neighboring gut cells are strongly connected through GJ and electrical coupling could synchronize endogenous pacemakers of individual cells.

## Results and Discussion

### Gut cell gap junctions

Microelectrode techniques allowed us to study nematode intestinal GJ in direct physiological experiments. (Fig. 1c,d,e,f) Fluorescent dyes were electrophoretically injected into gut cells via microelectrodes. Lucifer yellow CH diffuses in a matter of minutes from an initially injected cell to all intestinal cells (Fig. 1c, n = 4). At the same time, carboxyfluorescein fluorescent dye remained localized in the injected cell (Fig. 1d, n = 5). This observation indicates that individual cells within a chain are connected by selective intercellular channels.

All gut cells are strongly electrically connected (Fig. 1e,f) and the mean ± SEM coupling coefficient between two neighboring cells is 0.95 ± 0.012 (n = 10). The coupling coefficient between distant cells decreases in a geometrical progression. For example, for six-cell distance the coupling coefficient is about 0.75 that is close to the theoretical value of 0.95^6^ = 0.74.

Peters *et al*.^12^ demonstrated the presence of innexin/pannexin (INX-16) GJ molecules in nematode intestinal cells and their importance for the coordination of *C*. *elegans* DMP^14,15^. Now we have shown directly that nematode gut cells are linked tightly and form a functional electric syncytium.

### Membrane potential and defecation motor program cycling

**Figure 2:**
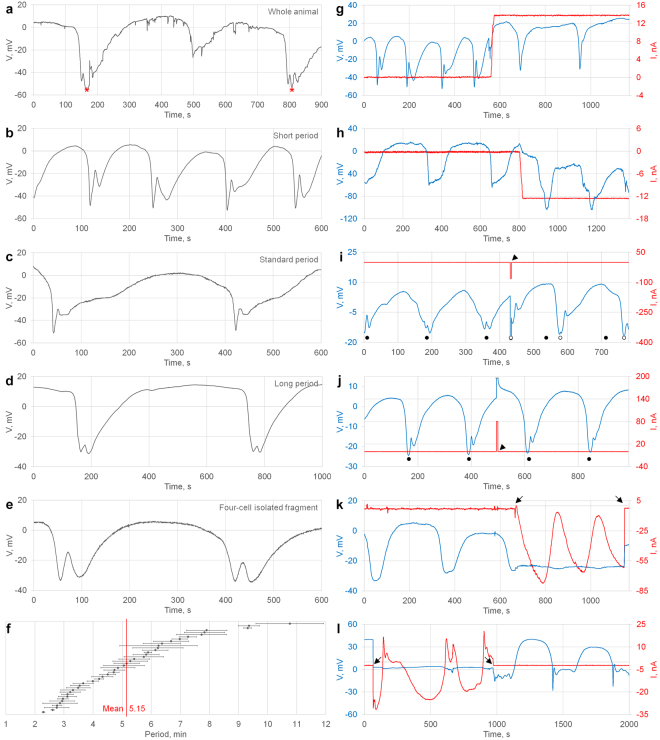
Rhythmical electrical activity in *H*. *megidis* nematode gut. (**a**) Intracellular recordings of the spontaneous voltage oscillations in a whole animal (asterisks indicate defecation expulsions) and (**b**,**c**,**d**,**e**) isolated gut preparations. Examples of (**b**) short, (**c**) medium and (**d**) long cycle periods. (**e**) Small (four-cell) isolated gut fragment. (**f**) DMP period variability expressed as mean (SD) for 41 experiments with minimum four DMP cycles recorded, ranked according to mean period length. (**g**,**h**,**i**,**j**,**k**,**l**) Intracellular two-electrode recordings (voltage – blue, current – red). (**g**) Continuous depolarization via current electrode injection increases the DMP period and (**h**) continuous hyperpolarization decreases the period. **i** Short hyperpolarizing current pulse (arrowheads) resets DMP rhythm while **j** depolarizing current pulse produces no effect. Black dots mark initial DMP period. White dots mark phase after reset. Effects shown in (**g**,**h**,**i**,**j**) were reproduced for at least three times. (**k**,**l**) Current cycling in two experiments with different voltage clamp levels (−25 mV and 0 mV). Arrows show on and off voltage fixation turning.

*H*. *megidis* DMP manifests in rhythmical changes of the membrane potential in gut cells (Fig. 2). Such oscillations could be recorded and analyzed by various electrophysiological techniques using intracellular microelectrodes. In most experiments, membrane potential oscillations in intestinal cells have an amplitude of about 60 mV and a period of about 5 minutes (Fig. 2a,b,c,d,e,f). The potential starts at the low positive depolarization level and transits to a rapid all-or-none hyper-polarization “action potential”, which later slowly tends back to previous values.

The mean period of recorded by electrophysiological methods oscillations are similar to the defecation cycle periodicity of the whole animal. The mean period between two observed pBoc, as the most identified characteristic of the defecation motor program, has the same length about 5 minutes (see Supplementary Notes and Fig. S1).

Unlike *C*. *elegans*, *H*. *megidis* is a parasite, so its cuticle is softer and allows the penetration of gut cells by the microelectrode in the whole worm (Fig. 2a) placed in agarose gel to restrain animal’s movement. Some noise is inevitable in such experiments due the residual nematode movement. During *in vivo* electrophysiological recordings defecation expulsion time corresponds to the rapid hyperpolarization phase (Fig. 2a). No substantial difference was detected between recordings from the isolated gut preparations (Fig. 2b,c,d), *in vivo* experiments (Fig. 2a) and isolated gut fragments with a small number of cells (Fig. 2e). They have approximately the same time-course and range of membrane potentials. The maximal observed oscillations amplitude was 87.5 mV, maximal period was more than 10 minutes (Fig. 2d), and the shortest period was about 2 minutes (Fig. 2b).

A steady depolarization of the gut cells slows down the DMP rhythm whereas hyperpolarization accelerates it (Fig. 2g,h). Short strong hyperpolarization current pulse (~−80 nA; 4 sec) can trigger extra all-or-none hyper-polarization action potential and resets the rhythm (Fig. 2i). Analogous depolarization current pulse (~+80 nA; 4 sec) has no effect on the DMP rhythm (Fig. 2j). Electrical activity in the nematode intestine cells resembles the action potentials generation in regular excitable tissues, neurons or myocytes. A common spike in excitable tissues is also triggered by the shift of the membrane potential beyond the certain threshold. Ongoing chain of spikes depends on the membrane potential and could be entrained with artificial current pulse. The same is true for *H*. *megidis* gut, but with reversed polarity of voltage and current.

By analogy, we may consider that the intestinal cell rhythmic electric potential generation is based on the cooperation of voltage gated ion channels in *H*. *megidis*. Nevertheless, in the isolated nematode gut electric current cycling persisted in two-electrode voltage clamp experiments where membrane potential was continuously maintained at steady voltage levels (Fig. 2k,l). Therefore, the rhythm observed in those experiments is not driven exclusively by the activity of plasma membrane voltage gated ion channels. According to these results, we propose that two distinct pacemakers are acting together to coordinate intestinal DMP, one based on plasma membrane channels and another based on intracellular molecular mechanisms.

### Gut cell electrical properties

**Figure 3:**
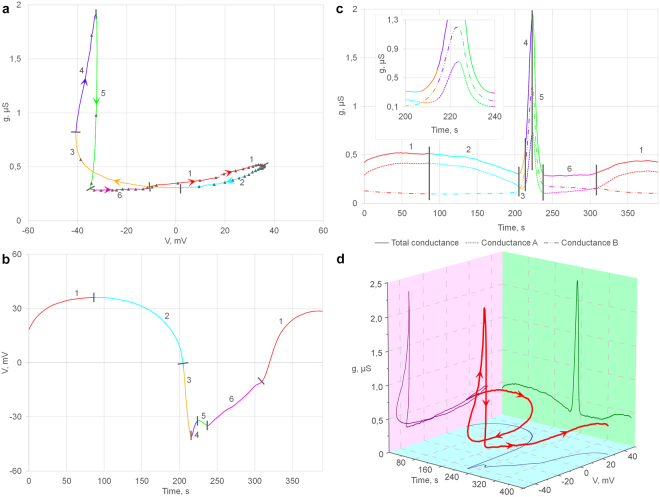
Gut cell membrane potential (*V*) and conductance (*g*) in DMP cycling (**a**,**b**,**c**) *g(V)*, *V(t)*, and *g(t)* dependences from one recorded DMP cycle. (**a**) In phase portrait dots are set in each 7 sec. The cycle is separated into 6 phases that differ in their *V* and *g* time course. (**c**) Membrane conductance (solid line) is divided in *g*_*a*_ (dotted line) and *g*_*b*_ (dash-dotted line) components according to the model (see text for details), time extended phases 3–5 are shown in inset. (**d**) *g*, *V*, *t* from a single gut cell DMP cycle were used to draw 3D trajectory plot (red). *g(V)* – magenta, *g(t)* – green and *V(t)* – blue are represented at projections. Arrows in (**a**,**d**) clarify the time course.

To understand DMP rhythmic generation process in details the cell membrane conductance (*g*) was measured in the time-course of the defecation cycle in an isolated gut preparation (n = 14) According to *V(t)*, *g(t)* and *g(V)* dependences DMP cycle was partitioned into six (1–6) components (Fig. 3a,b,c). For the reason that an increase of conductance could be associated either with hyperpolarization or depolarization depending on the cycle phase, at least two distinct types of ion conductance are needed to explain a phase portrait (*g(V)* curve) in the gut oscillation. (Fig. 3a).

To evaluate the simplest model of this process the observed membrane conductance (*g*_*r*_) was separated into two components: one (*g*_*a*_) for a cation with extracellular concentration that is much higher than intracellular (presumably Na^+^ or Ca^2+^), and one (*g*_*b*_) for a cation with an intracellular concentration that is much higher than extracellular (presumably K^+^). Membrane reverse potentials for these components were arbitrary set at *V*_*a*_ =  + 70 mV; *V*_*b*_ = −100mV respectively. The membrane conductance is additive (1)

1 $${g}_{r}={{\rm{g}}}_{a}+{{\rm{g}}}_{b}$$

With those assumptions, total membrane potential *V*_*r*_ can be calculated according to formula (2)^16^:

2 $${V}_{r}=\frac{{V}_{a}{g}_{a}+{{\rm{V}}}_{b}{g}_{b}}{{g}_{a}+{{\rm{g}}}_{b}}$$

From these two equations, *g*_*a*_ and *g*_*b*_ can be expressed as (3) and (4):

3 $${g}_{a}=\frac{({V}_{r}-{{\rm{V}}}_{b}){g}_{r}}{{V}_{a}-{{\rm{V}}}_{b}}$$

4 $${g}_{b}=\frac{({V}_{r}-{{\rm{V}}}_{a}){g}_{r}}{{V}_{b}-{{\rm{V}}}_{a}}$$

The graphs shown in Fig. 3a,b,c imply that the slow depolarizing “bump” (phase 1 and 2) is shaped predominantly by *g*_*a*_ whereas the shorter hyperpolarization spike (phase 3–6) is formed by the superposition of both *g*_*a*_ and *g*_*b*_. The shape of *g*_*b*_ *(t)* function is simple – with one peak while *g*_*a*_ *(t)* has two peaks (Fig. 3c). Therefore, we considered that *g*_*a*_ could represent combined permeability of *g*_*a1*_ and *g*_*a2*_ for two distinct ion channels.

**Figure 4:**
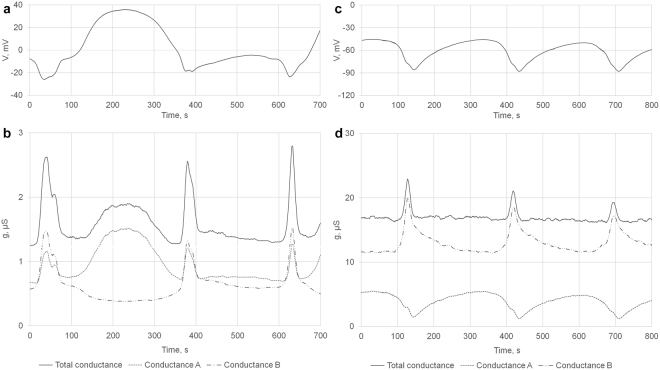
Unusual patterns of defecation motor program oscillations. (**a**,**b**) In the second cycle depolarizing “bump” dependent on *g*_*a1*_ is missing. (**c**,**d**) Rhythm generated by *g*_*a*_ displayed only one, apparently *g*_*a2*_ peak. As in Fig. 3c for (**b**,**d**) membrane conductance (solid line) is divided in *g*_*a*_ (dotted line) and *g*_*b*_ (dash-dotted line) components according to the model.

This consideration is supported by several examples of DMP recordings of unusual rhythm generation with *g*_*a1*_ or *g*_*a2*_ component missing (Fig. 4). In Fig. 4a, b depolarizing bump (*g*_*a1*_) is missing in one cycle while flanking hyperpolarization events appeared to be normal. In the other case, (Fig. 4c,d) the rhythm generated by *g*_*a*_ displayed only one, apparently *g*_*a2*_ peak.

### Mechanisms of defecation motor program generation

The nematode gut contains no neuronal or muscle cells and the CPG resides in the intestine itself as was shown for *C*. *elegans* ^9,10,17^ and *H*. *megidis* isolated gut experiments. This CPG rhythm is based on the intrinsic properties of individual cells as follows from *C*. *elegans* experiments. It was shown, that gut cells were oscillating independently while recorded in animals with disrupted GJ^12^. A. Estevez and K. Strange also demonstrated cyclic activity in individual embryonic gut cells in culture^18^. *H*. *megidis* data is consistent with this conclusion. Different parts of the isolated intestine including small (3–8 cells) gut fragments behave similar to the whole gut preparations.

**Figure 5:**
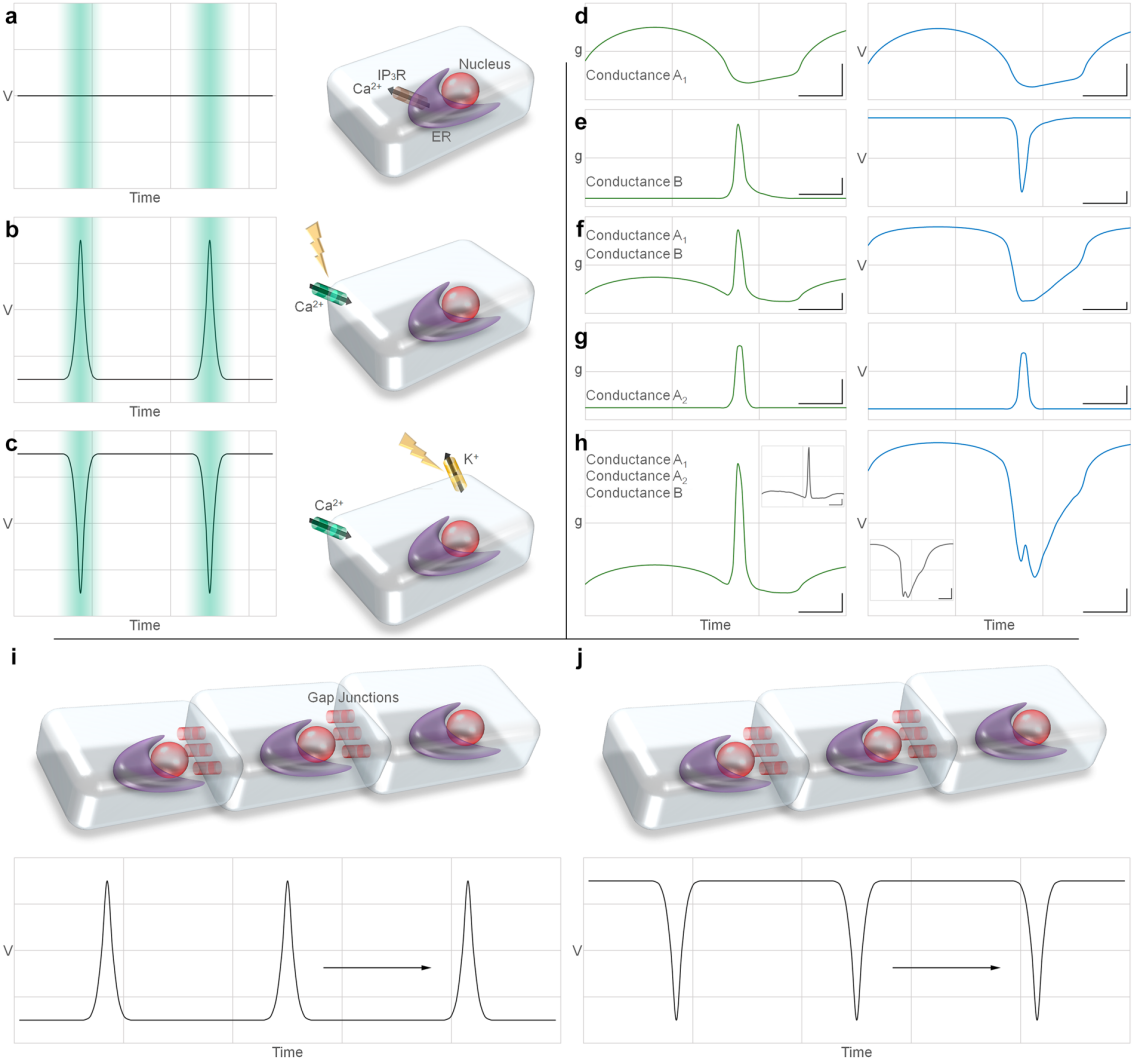
Overview of defecation motor generation and propagation. (**a**,**b**,**c**) Membrane potential (*V*) and Ca^2+^ wave (cyan shading) for three theoretical mechanisms of DMP generation. (**a**) IP_3_R dependent ER Ca^2+^ release. *V* is constant. (**b**) Voltage gated Ca^2+^(Na^+^) spike. (**c**) Voltage gated hyperpolarization K^+^ action potential drags Ca^2+^ through leak channels. (**d**,**e**,**f**,**g**,**h**) simple model reconstruction of *g(t)* (left, green), and *V(t)* (right, blue) dependences from three types of plasma membrane conductance closely matched to experimental data. Calibration: 10 sec; 0.2 µS (left); 15 mV (right). Insets in (**h**) demonstrate actual experimental curves. (**i**) In nervous and muscle tissue, depolarization spike can spread through a chain of excitable cells connected via GJ. (**j**) In nematode gut similar signal propagation relies on hyperpolarization action potential.

The periodic wave of intercellular Ca^2+^ concentrations was used as a hallmark of DMP cycling in most of *C*. *elegans* studies^11,12,19–21^. Unfortunately, due to the gut strong auto florescence we were not currently able to apply Ca^2+^ imaging in *H*. *megidis* and consider it presence in this species by analogy to evolutionally related *C*. *elegans*. Theoretically, three fundamentally different mechanisms can recruit ion channels to enable membrane potential and Ca^2+^ oscillations (Fig. 5a,b,c).

### Intracellular endoplasmic reticulum (ER) oscillations

ER calcium release mediated through inositol 1,4,5-trisphosphate and its receptor *itr-1* is thought to be the most important element in the intestinal cells Ca^2+^ oscillator in *C*. *elegans* studies^13,22–24^. Indeed, *itr-1* mutants show long defecation cycle periods or no defecation behavior, while overexpression of this gene results in short defecation cycle periods^18^. The persistence of the DMP rhythmicity under the voltage clamp conditions in *H*. *megidis* is consistent with the crucial role of IP_3_ receptor in nematode DMP pacemaker. Potentially Ca^2+^ oscillations may proceed entirely on intracellular machinery that will rhythmically liberate Ca^2+^ from ER and resorb it back with no effect on the membrane potential (Fig. 5a).

### Depolarizing spike

The most common mechanism for membrane potential and Ca^2+^ oscillations in excitable cells is based on voltage gated channels activity that generates action potentials named spikes^25^. In such case basic cell membrane potential is sustained at negative level and transient Na^+^ and/or Ca^2+^ currents briefly depolarize membrane and conduct Ca^2+^ influx. (Fig. 5b).

### Hyperpolarizing spike (“pit”)

The shape and electrical properties of the membrane potential oscillations in the *H*. *megidis* gut cells resemble overturned spikes in neurons and myocytes. It shifts from basic depolarized state to brief hyperpolarization (Fig. 5c). The frequency of these oscillations depends on membrane potential and could be entrained by electrical pulses similar to usual spikes. In this case, transient hyperpolarization can be caused by K^+^ channel opening. Ca^2+^ is pulled in the cell by resulting voltage gradient via Ca^2+^ leak channels (“trees make the wind blow”).

We suggest that all three above-mentioned mechanism are important for nematode DMP and act together.

The simplest model shown in Fig. 5d,e,f,g,h includes only three plasma membrane ion channels with *g*_*a1*_, *g*_*a2*_ and *g*_*b*_ conductance. *g*_*a1*_ can be attributed to Na^+^ channels. In Fig. 5d *g*_*a1*_ (shown in left) is set similar to experimental data and generated calculated membrane potential voltage shown in right. Hypothetical *g*_*b*_ is probably caused by K^+^ channel. Its time-course was set as depicted in Fig. 5e left. Resulting voltage curve is shown in Fig. 5e right. The superposition of *g*_*a1*_, *g*_*b*_ results in more realistic *g(t)* and *V(t)* plots (Fig. 5f). *g*_*a2*_ can represent a putative membrane Ca^2+^ channel. *g*_*a2*_ produce brief Ca^2+^ influx during hyperpolarizing spike (pit) phase. The superposition of all three *g*_*a1*_, *g*_*a2*_, *g*_*b*_ conductance and calculated voltage plots closely resemble actual recordings (insets in Fig. 5h) including distinctive small peak at phase 4–5 on Fig. 3a,b,c.

*g*_*a1*_, *g*_*a2*_, *g*_*b*_ could be associated with already known Na^+^, Ca^2+^ and K^+^ channels in *C*. *elegans*. One sodium channel was shown to contribute to *C*. *elegans* DMP cycling. Mutants in *flr-1* show very short and unstable defecation periods^26^. This gene is expressed in intestine cells and encodes an ion channel belonging to the degenerin (DEG) amiloride sensitive epithelial sodium channel^27^. For hyperpolarization “pit” action potential voltage gated K^+^ channel could be viewed as analogous to Na^+^ or Ca^2+^ voltage gated channels implicated in usual spikes. Voltage gated K^+^ channels are well known in different organisms, including nematodes although their function was considered different^28^. Alternative mechanism to produce hyperpolarizing spike can utilize calcium-activated potassium (K_Ca_) channels. We can expect that Ca^2+^ influx to cytoplasm caused by natural or artificial hyperpolarization can activate K_Ca_ in positive feedback mode. Among many *C*. *elegans* K^+^ channels two KNQ (*kqt-2* and *kqt-3*) channels were implicated in intestinal cells DMP function^9^. Several Ca^2+^ selective ion channels implicated in *C*. *elegans* DMP belong to TRPM channel family: *gtl-1*, *gtl-2* and *gon-219* ^29^.

More accurate association of *H*. *megidis* DMP machine to specific proteins and pathways predicted in *C*. *elegans* studies is beyond the scope of the present brief report and will require intense pharmacological approach and direct Ca^2+^ concentration measurements.

In *C*. *elegans* intercellular GJ channels formed by innexin/pannexins family proteins were shown to establish synchronization of gut cells activity^19^. This is confirmed by our direct physiological study with both dye injection and electrical coupling experiments. One important refinement to intercellular DMP synchronization consider that intestinal cells are voltage sensitive. The calcium signals in *C*. *elegans* ^12,19^ initiate in the posterior and nearly simultaneously at the anterior. The time period between these two events is not sufficient for any kind of chemical message (IP_3_ etc.) to spread from one end to the other. Therefore, voltage shift in one cell can trigger next cell activity by direct electrical coupling even without direct intercellular diffusion of signaling molecules (as IP_3_ or Ca^2+^).

Although endoderm is not considered to be excitable tissue, self-amplifying and self-propagating electrical wave in nematode gut reminds action potential in excitable cells. Figure 5g,h schematically compare DMP wave propagation in nematode gut with in a chain of excitable cells like heart or annelid septal axon^30^. The similarity of these phenomena calls for a reevaluation of the excitable cells definition.

## Materials and Methods

*H*. *megidis* nematodes^31^ were obtained from moth larvae hosts in 3–5 days after L1 infection^32^. All studies were carried out in Hanks solution (0.137 M NaCl, 5.4 mM KCl, 0.25 mM, Na_2_HPO_4_, 0.44 mM KH_2_PO, 1.3 mM CaCl, 1.0 mM MgSO_4_, 4.2 mM NaHCO_3_) at 20 °C.

The nematode guts were isolated using fine forceps and scissor, placed in Petri dishes with solid 1.5% agarose based on Hanks solution at the bottom and covered by 1 mm layer of liquid Hanks solution.

Axoclamp 2B (Axon Instruments) amplifiers coupled with glass microelectrodes filled in with 2 M KCl were used for records. The resistance of electrodes was within the 10–20 MΩ range. The recorded data was saved and further analyzed using a PC with a DigiData 1200 series, ADC, pCLAMP program (Molecular Devices, United States), Microsoft Office tools and Origin 9.1.

For fluorescent dye injection, one of the electrodes was filled in with 0.1 M KCl solution containing 3% carboxyfluorescein or 0.1 M LiCl solution containing 3% Lucifer yellow CH. The dye injection in a single gut cell was carried out using a negative current of 10 nA for 5 min. Guts injected with fluorescent dyes were studied using an Olympus BX51 fluorescent microscope equipped with an Olympus XC10 camera and a set of filters optimized for fluorescein or Lucifer yellow. All reagents were obtained from Sigma-Aldrich.

To measure cell membrane resistance (or conductance) short (0.5 sec.) repetitive current pulses were injected every 1.5 sec. (Fig. S2a). Cell membrane resistance or conductance were calculated according to Ohm’s law. To smooth the data curves (Fig. S2c) dots were sampled only at inter-steps time.

## Electronic supplementary material

Supplementary Notes and Figures
