# Supplementary material for: Electrophysiology of the rhythmic defecation program in nematode Heterorhabditis megidis
Source: Sci Rep. 2017 Dec 19;7:17834. doi: 10.1038/s41598-017-18118-y (PMC5736584; doi:10.1038/s41598-017-18118-y)
Supplement: Supplementary file 1 — Supplementary Notes and Figures [file 41598_2017_18118_MOESM1_ESM.pdf]

Supplementary information

Electrophysiology of the rhythmic defecation program in nematode *Heterorhabditis megidis*

Victor P. Kuznetsov*,[1,2] Georgy A. Slivko-Koltchik,[1,2] Dmitry A. Voronov,[1,3] and Yuri V. Panchin[1,3],

[1]Kharkevich Institute for Information Transmission Problems, Russian Academy of Sciences, Moscow 127994, Russian Federation

[2]Faculty of Bioengineering and Bioinformatics, Lomonosov Moscow State University, Moscow 119991, Russian Federation

[3]A.N. Belozersky Institute of Physico-Chemical Biology Moscow State University Moscow 119992 Russian Federation

*scikvp@gmail.com

Supplementary Notes

The first generation of adult hermaphrodites of entomopathogenic rhabditid nematode *Heterorhabditis megidis* Poinar, Jackson and Klein, 1987[1] were obtained from the laboratory of Dr. S.E. Spiridonov (A.N. Severtsov Institute of Ecology and Evolution, Russian Academy of Science). In our experiments, we used animals with the body length from 1 to 4 mm. The intestine comprises approximately 90% of the body length. The number of *H. megidis* intestinal cells were estimated as 24-28 (for comparison, the intestine of *C. elegans* consists of 20 cells[2]). They are arranged similar to the intestinal cells in *C. elegans*. The intestinal cell borders are clearly visible. Still, the precise counting of gut cells is complicated. Intact animal gonads are nontransparent and strongly decrease the field of view, while in the isolated gut preparations, some cells can be missing. As in *C. elegans*[3], the gut cells of *H. megidis* contain multiple nuclei (Fig. S3), therefore their staining is not helpful for cell counting.

Only about a quarter of nematodes taken to the experiment appear to show membrane potential oscillations or DMP cycling. The mean (SD) period of electrical membrane oscillation was close in isolated preparations (5.14 (2.08) minutes n=41) and in whole animals (7.17 (1.863) minutes n=6). pBoc is a well identified contraction, while aBoc is not. The period between two pBoc measured in intact animals was 5.84 (0.94) minutes n=7 (Fig. S1). The contraction of the *H. megidis* tail length lasts approximately 6 seconds.

Supplementary Figures

Supplementary Fig. s1

pBoc in intact *H. megidis* nematode. Panels **a** and **b** show two different nematodes during pBoc. Each figure represents superimposition of the two photographs of the same animal with the 6-second interval. Starting phase is outlined in cyan and contraction (6 second later) is outlined in red.

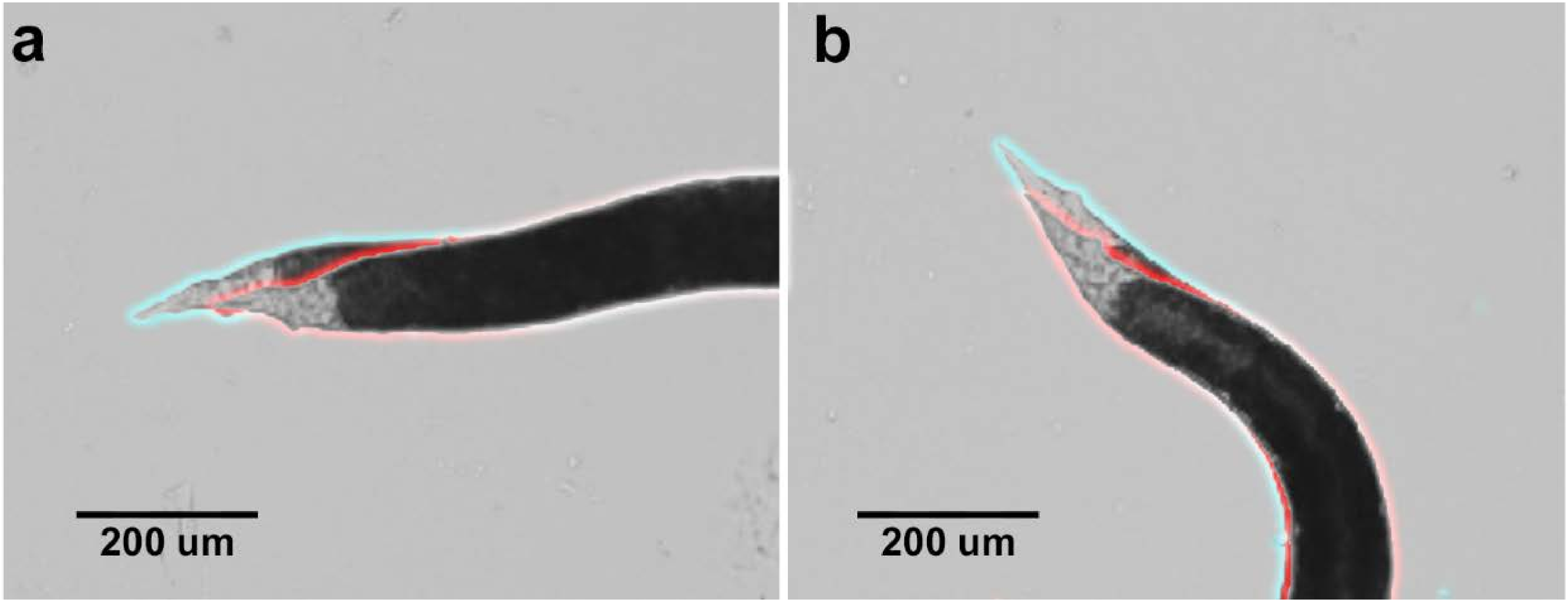

Supplementary Fig. s2

Gut cell membrane conductance measurements. a Five second fragment from full current and voltage plot. Small current pulses lead to voltage changes in the cells of isolated gut preparations. Raw b and smoothed c voltage plot. Current – red; voltage blue.

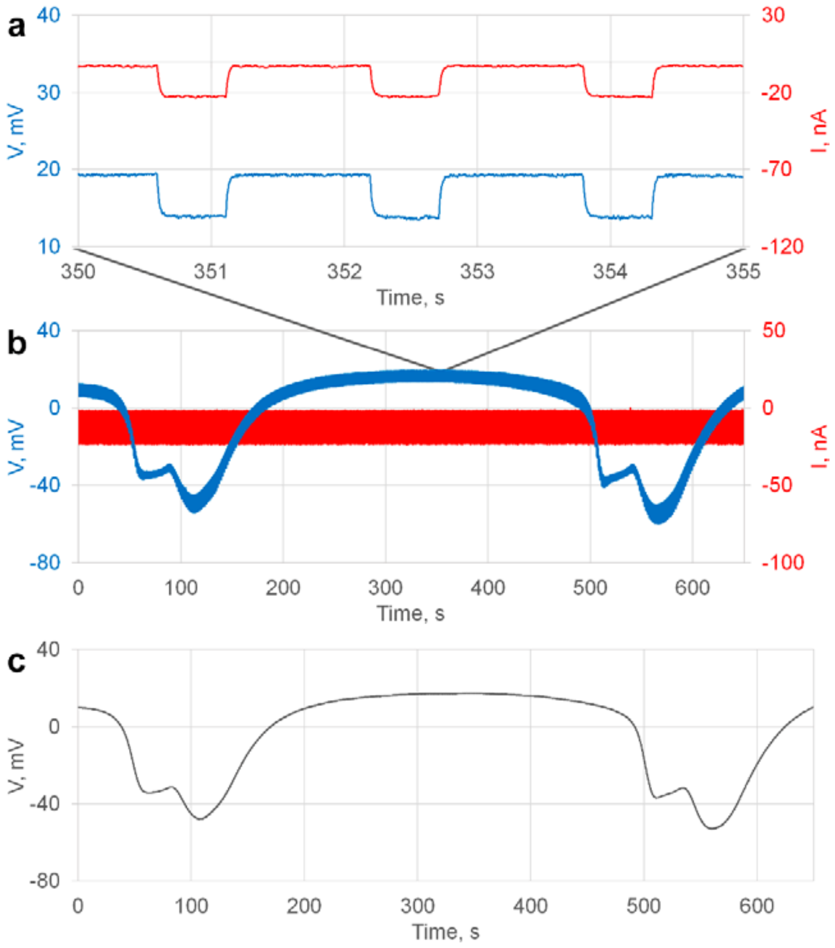

Supplementary Fig. s3

Isolated *H. megidis* intestine preparation with ethidium bromide staining. Panels **a** and **b** are different magnifications of the one preparation in red long-pass WG fluorescence cube. Each cell has multiple nuclei. Nuclei are marked by white arrows. Blue arrows point to cell borders.

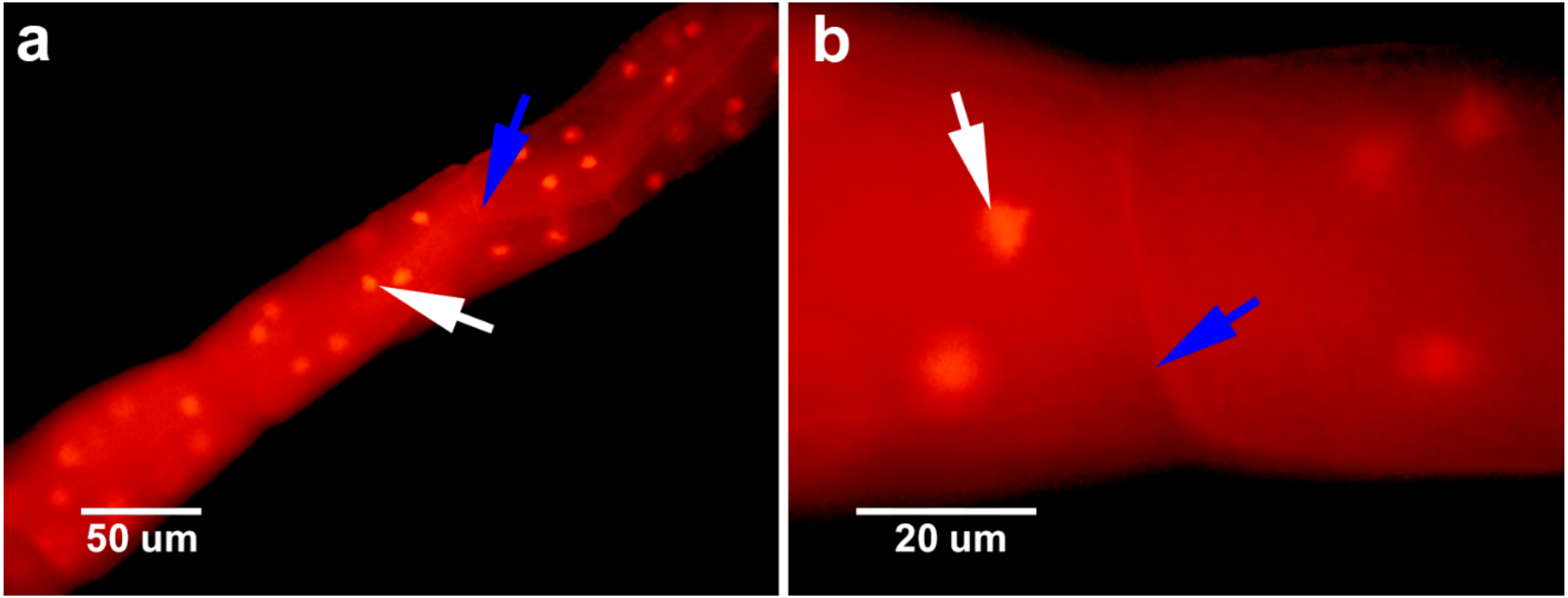

Supplementary Fig. s4

No significant difference in oscillation period was observed between large (more than 3mm in length) and small (less than 3mm in length) nematodes. The mean±SEM period for large intestine preparations (blue) is 4.85±0.62 minutes (n=13), the mean±SEM period for small intestine preparations (orange) is 4.67±0.56 minutes (n=6).

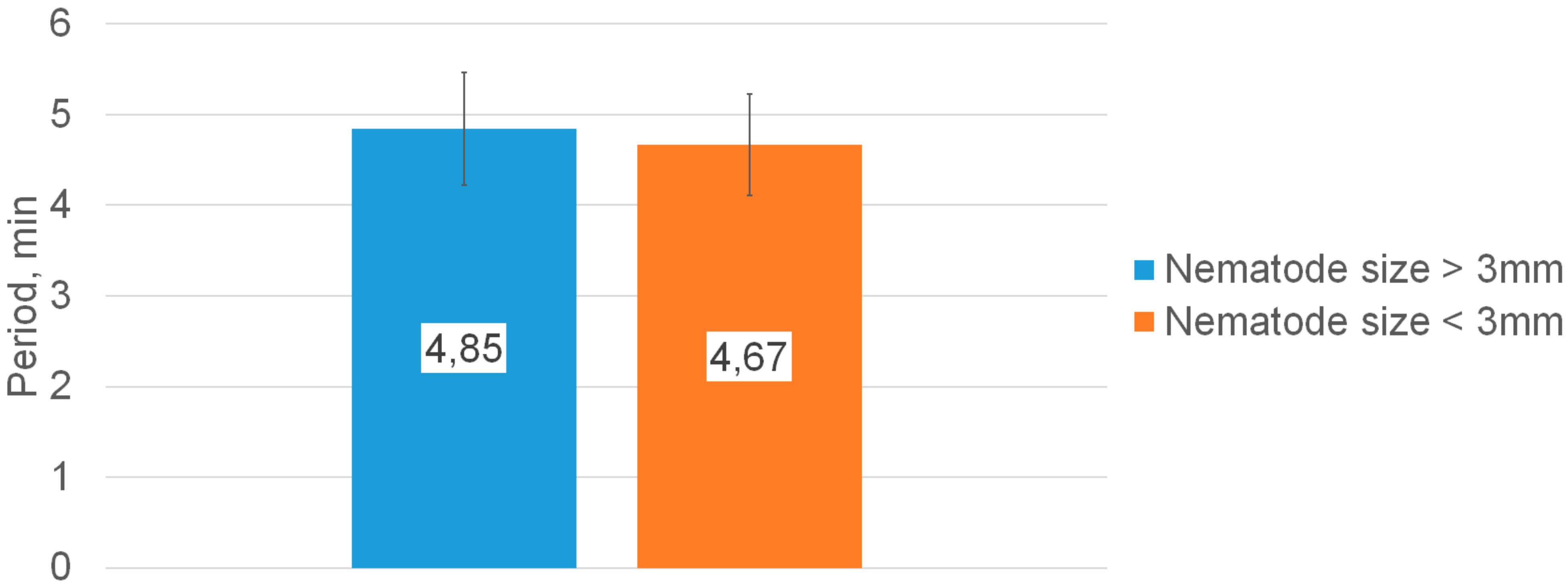

Supplementary Fig. s5

There is no significant difference in oscillation period between three types of preparations. Mean±SEM values for anterior (blue), posterior (orange) and fully isolated (grey) types of preparation are 4.5±0.87 min (n=4), 4.88±0.48 minutes (n=8), 4.6±0.68 minutes (n=5) accordingly.

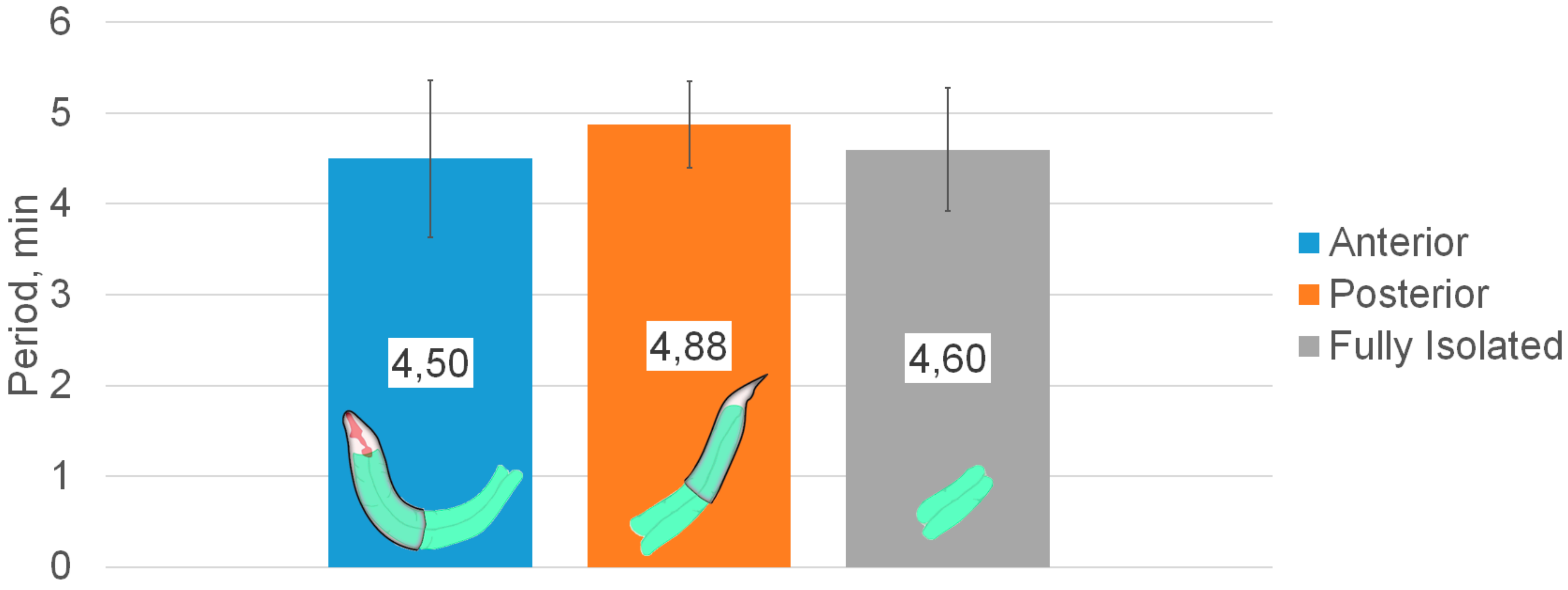

Supplementary Fig. s6

Voltage pattern of *H. megidis* membrane potential oscillations. To compare recordings from different experiments, the typical oscillation cycles from 28 experiments were analyzed. The period and amplitude of each case were normalized to 100% and the resulting normalized graphs were subjected to further statistical analysis.

Blue line represents the mean time course of normalized membrane potential oscillations. Blue dots indicate the lower amplitude value that was taken as the starting point in the each recording. Red dots indicate the local extrema. Bars around red dots indicates values of SD for time and amplitude calculated for normalized data (the maximal point was established as 100% for the each experiment that is why only time depending SD bar is given).

In addition, two bars with real (non-normalized) values are given. Blue bar is the mean (SD) amplitude 46.8 (7.3) mV and gray bar is the mean (SD) period of membrane oscillation 5.3 (2.4) min.

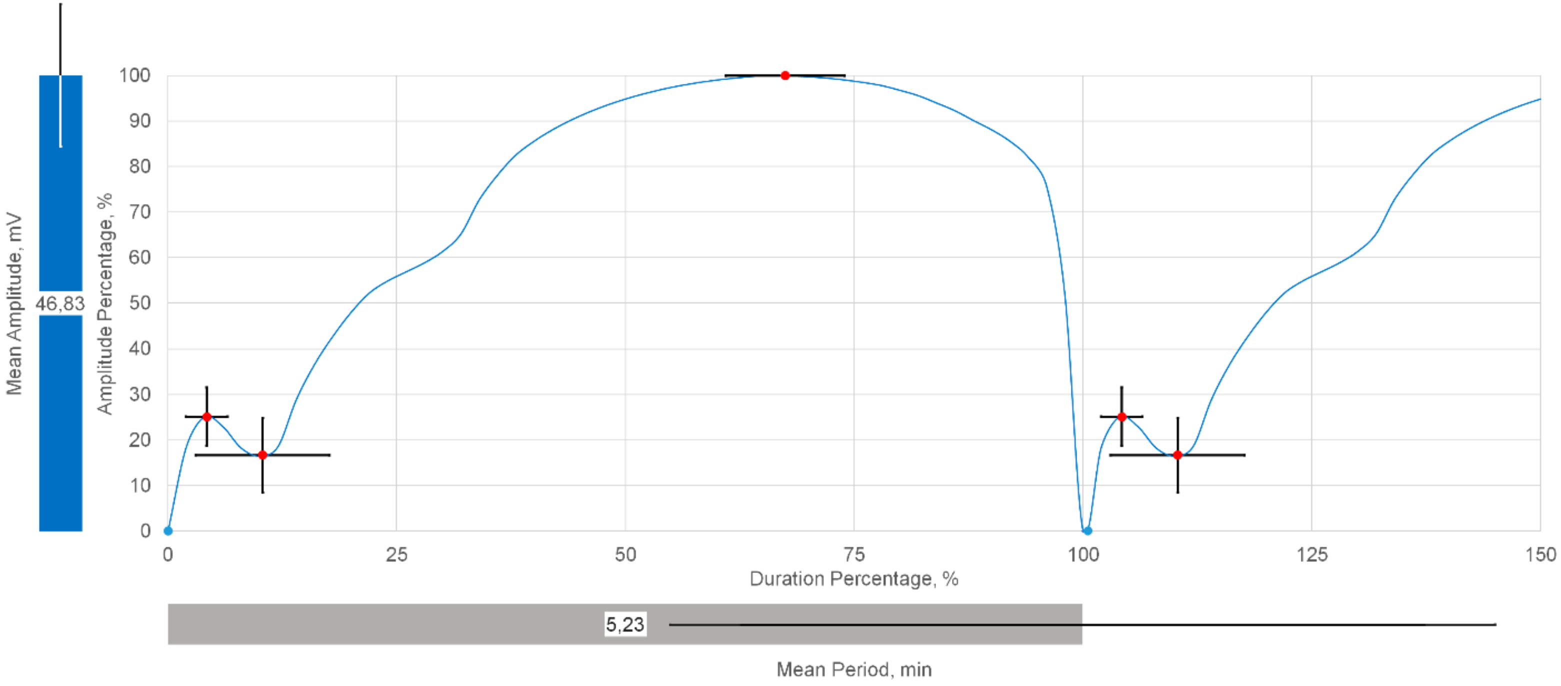

Supplementary references

1. Poinar, G.O., Jr., T. Jackson, M. Klein. *Heterorhabditis megidis* sp. n. (*Heterorhabditidae*: *Rhabditida*) parasitic in the Japanese beetle, Popillia japonica (*Scarabaeidae*: *Coleoptera*), in Ohio. *J Parasitol.* **54**, 53-59 (1987)

2. Sulston, J. E., Schierenberg, E., White, J. G. & Thomson, J. N. The embryonic cell lineage of the nematode Caenorhabditis elegans. *Dev. Biol.* **100,** 64–119 (1983).

3. Wolf, M., Nunes, F. & Paul, R. J. Coordinates, DNA content and heterogeneity of cell nuclei and segments of the Caenorhabditis elegans intestine. *Histochem. Cell Biol.* **124,** 359–367 (2005).
